# Crystal Transition Behavior and Thermal Properties of Thermal-Energy-Storage Copolymer Materials with an *n*-Behenyl Side-Chain

**DOI:** 10.3390/polym11091512

**Published:** 2019-09-17

**Authors:** Yuchen Mao, Jin Gong, Meifang Zhu, Hiroshi Ito

**Affiliations:** 1 Department of Polymer Science and Engineering, Graduate School of Organic Materials Science, Yamagata University, 4-3-16 Jonan, Yonezawa, Yamagata 992-8510, Japan; maoyc91@gmail.com; 2 State Key Laboratory for Modification of Chemical Fibers and Polymer Materials, College of Materials Science and Engineering, Donghua University, Shanghai 201620, China; 3 Department of Mechanical Systems Engineering, Graduate School of Science and Engineering, Yamagata University, 4-3-16 Jonan, Yonezawa, Yamagata 992-8510, Japan

**Keywords:** crystal transition behavior, thermal property, thermal energy storage, copolymer, suspension polymerization, light-induced polymerization

## Abstract

In this paper, we synthesized MC(BeA-*co*-MMA) copolymer microcapsules through suspension polymerization. The pendent *n*-behenyl group of BeA is highly crystalline, and it acts as the side-chain in the structure of BeA-*co*-MMA copolymer. The highly crystalline *n*-behenyl side-chain provides BeA-*co*-MMA copolymer thermal-energy-storage capacity. In order to investigate the correlation between the thermal properties and crystal structure of the BeA-*co*-MMA copolymer, the effects of monomer ratio, temperature changing and the changing rate, as well as synthesis method were discussed. The monomer ratio influenced crystal transition behavior and thermal properties greatly. The DSC results proved that when the monomer ratio of BeA and MMA was 3:1, MC(BeA-*co*-MMA)3 showed the highest average phase change enthalpy Δ*H* (105.1 J·g^–1^). It indicated that the *n*-behenyl side-chain formed a relatively perfect crystal region, which ensured a high energy storage capacity of the copolymer. All the DSC and SAXS results proved that the amount of BeA had a strong effect on the thermal-energy-storage capacity of the copolymer and the long spacing of crystals, but barely on the crystal lamella. It was found that MMA units worked like defects in the *n*-behenyl side-chain crystal structure of the BeA-*co*-MMA copolymer. Therefore, a lower fraction of MMA, that is, a higher fraction of BeA, contributed to a higher crystallinity of the BeA-*co*-MMA copolymer, providing a better energy storage capacity and thermoregulation property. ST(BeA-*co*-MMA) copolymer sheets with the same ingredients as microcapsules were also prepared through light-induced polymerization aiming at clarifying the effect of the synthesis method. The results proved that the synthesis method mainly influenced the copolymer chemical component, but lightly on the crystal packing of the *n*-behenyl side-chain.

## 1. Introduction

A latent heat storage system is a highly efficient and environmentally friendly means to use residual heat, recovering waste heat and to store/release thermal energy for saving energy. Phase change materials (PCMs) are considered to be among the most reliable latent heat storage and thermoregulation materials, which work by absorbing or releasing the enthalpy of phase changes in certain temperature ranges. PCMs have been successfully utilized in aerospace and aviation, solar engineering, building air conditioning, insulating clothing and textiles, preservation and environmental temperature-control since their developments in the 1980s [1,2,3,4,5,6].

Polymeric PCMs transform from one semi-crystalline state into another semi-crystalline state, or an amorphous state, with the advantages of small volume change, high energy-storage density, great design flexibility and form-stable at a temperature above the melting point [7,8,9]. Polymeric PCMs also benefit from less phase segregation and good physical-chemical stability. These features give them perfect thermal durability and cycling stability against thermal degradation required for a long-term service [10,11,12,13]. Comb-like polymers, with a crystalline long *n*-alkane side-chain attached to a main-chain, are known to pack into a layered structure with alternating side-chain crystal regions at room temperature [14,15,16,17]. In our group, much of the work revolves about crystalline copolymer microcapsules and gels having a chemical structure with an *n*-alkane crystalline side-chain. The behenyl acrylate (BeA) consisted of an *n*-behenyl chain with 22 methylene units and a vinyl group was used as the phase change monomer to synthesize P(BeA-*co*-MMA) copolymer microcapsules with methyl methacrylate (MMA) through suspension polymerization [18]. Furthermore, we have developed crystalline gels with high toughness and high flexibility, as well as other functions such as shape memory, humidity regulation, thermal expansion and thermal energy storage [19,20,21,22,23,24]. We have confirmed that these copolymers can form crystal regions at room temperature to provide thermoregulation properties, and their application potential as form-stable solid–solid PCMs are greatly expected. 

**Figure 1:**
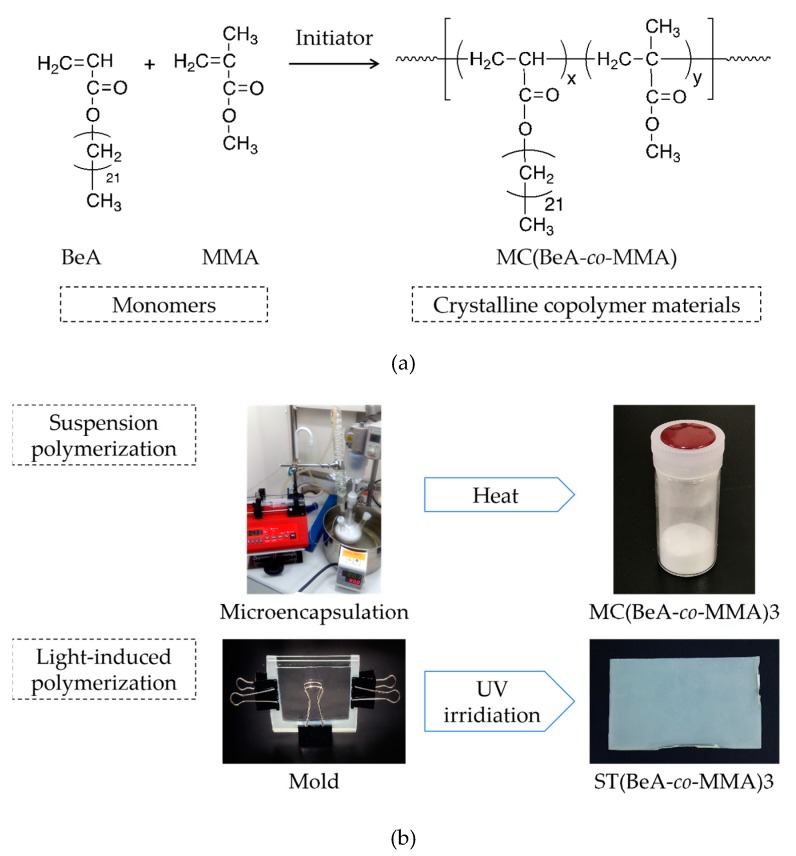
Synthesis schemes of thermoregulation MC(BeA-*co*-MMA) copolymer (**a**), and the microcapsules and sheets prepared through suspension and light-induced polymerization, respectively (**b**). The phase transition between the crystal phase and the isotropic phase promises the energy storage capacity.

For the crystalline polymers, crystallization controls the assembly of ordered crystalline structures from molecular chains, which determines the basic physical properties of crystalline polymer materials [25]. Therefore, in this paper, we aim to investigate the phase transition behavior between the crystal phase and the isotropic phase of the thermal-energy-storage copolymer materials with a crystalline *n*-behenyl side-chain. A series of copolymer microcapsules were synthesized from BeA and MMA, shorted as MC(BeA-*co*-MMA). Polymethyl methacrylate (PMMA) is a tough and rigid plastic having pendent methyl groups (–CH_3_), which prevent the polymer chains from rotating freely around a carbon–carbon bond and packing closely in a crystal pattern. The introduction of MMA would change the chain flexibility and segment motion. Moreover, copolymer sheets with the same ingredients as copolymer microcapsules, shorted as ST(BeA-*co*-MMA), were synthesized as comparison samples through light-induced polymerization [20]. The synthesis schemes of the MC(BeA-*co*-MMA) copolymer are shown in Figure 1a, and the microcapsules and sheets prepared through suspension and light-induced polymerization, respectively, are presented in Figure 1b. The effects of monomer ratio and synthesis method on the crystal transition behavior and thermal properties were studied.

## 2. Materials and Experiments

### 2.1. Materials

Monomer BeA was provided by Shin-Nakamura Chemical Co., Ltd., Wakayama, Japan. Monomer MMA, thermal initiator 2,2′-azobis (2-methylpropionitrile) (AIBN), photo initiator benzophenone (BP) and methanol were purchased from Wako Pure Chemical Industries, Ltd., Tokyo, Japan. Polyvinyl alcohol (PVA) provided by Kuraray Co., Ltd., Okayama, Japan, was used as an emulsifier. All the above reagents were used without further purification.

### 2.2. Preparation of Copolymer Microcapsules

Copolymer microcapsules were synthesized by free radical suspension polymerization in an emulsion system with an oil-soluble initiator. Random copolymerization was carried out with varying monomer mass ratios between BeA and MMA in a similar manner as we reported before [18]. Crystalline copolymer microcapsules MC(BeA-*co*-MMA)1, MC(BeA-*co*-MMA)3 and MC(BeA-*co*-MMA)5 with monomer mass ratios of two associated monomers from 1:1, 3:1 to 5:1 were synthesized (listed in Table 1). Heat-initiated poly(methyl methacrylate) and poly(behenyl acrylate), named MC(MMA) and MC(BeA), respectively, were prepared and evaluated too.

### 2.3. Preparation of Copolymer Sheets

For a comparison to microcapsules, we synthesized copolymer sheets with an *n*-behenyl side-chain using the same ingredients with the monomer mass ratios of BeA to MMA at 1:1, 3:1 and 5:1, marked ST(BeA-*co*-MMA)1, ST(BeA-*co*-MMA)3 and ST(BeA-*co*-MMA)5, respectively (listed in Table 1). The reactive uncured liquid resin consisted of BeA, MMA and photo initiator BP. BP was dissolved in MMA firstly, and melted BeA was added to make a homogeneous resin. Then, the liquid resin was injected into a mold with 1 mm thickness (Figure 1b), and cured under UV irradiation for 48 h at 30 °C. The UV lamp type, power and wavelength are the key factors for the UV irradiation process. The UV lamps with a peak wavelength of 365 nm were purchased from Toshiba Lighting & Technology Corporation (Yokosuka, Japan). The UV intensity varies depending on the irradiation distance. The intensity of UV reaching samples ranged from 200 to 300 μW·cm^−2^. Light-induced poly(methyl methacrylate) and poly(behenyl acrylate) sheets, named ST(MMA) and ST(BeA), were prepared in a similar manner.

All the thermal-energy-storage copolymer microcapsules and sheets with a crystalline *n*-behenyl side-chain synthesized in this work are listed in Table 1.

### 2.4. Characterization of Copolymer Microcapsules and Sheets

#### 2.4.1. Differential Scanning Calorimetry (DSC)

The thermal properties and phase transition behavior of copolymers were investigated using a differential scanning calorimeter (Q-2000, TA instruments Japan Inc., Tokyo, Japan) operating under a nitrogen flow. Samples of about 5 mg were heated to 80 °C at a heating rate of 5 °C·min^–1^, and held for 1 min to eliminate the thermal prehistory. Then, samples were cooled down to 0 °C with various cooling rates for measuring their crystal behavior. After holding for 1 min, samples were reheated up to 80 °C at the same cooling rate. Premium hermetic pans (TA Instruments Tzero #901683.901, TA instruments Japan Inc., Tokyo, Japan) were used for the measurements.

#### 2.4.2. Small-Angle X-ray Scattering (SAXS)

The crystal transition behavior and crystal structures of copolymer microcapsules were analyzed using temperature-variable, small-angle X-ray scattering (Nano-Viewer, Rigaku Corporation, Akishima, Japan) with a nickel-filtered Cu-*K*α radiation (wavelength λ = 1.5405 Å), and a high-speed two-dimensional imaging plate detector (Pilatus3 R 100K, Rigaku Corporation, Akishima, Japan). In order to detect and analyze the transition behavior of the side-chain and copolymer skeleton in the crystallization process, the camera distance was set to 0.1 m. The effective detection range can expand to 2*θ* = 30°. All measurements were carried out within the exposure time of 5 min and at a heating/cooling rate of 0.5 °C·min^–1^. The heating–cooling cycles of microcapsules were taken with the temperature ranging from 35 to 70 °C on a hot stage (FP82HT hot stage connected to FP90 central processor, Mettler Toledo, Tokyo, Japan). Diffraction diagrams recorded on the flat imaging plate detector by the beam-transmission technique were converted to the one-dimensional profile against diffraction angle 2*θ* through Igor Pro 7 (WaveMetrics Inc., Lake Oswege, OR, USA) with a special add-in progress. The interplanar crystal spacing *d* was calculated by the following formula:

(1) $$d=\frac{\lambda }{2\sin\theta }$$

#### 2.4.3. Wide-Angle X-ray Scattering (WAXS)

To determine the crystal structure and crystallinity of copolymer materials, wide-angle X-ray scattering was performed on an X-ray diffractometer (Ultima IV, Rigaku Corporation, Akishima, Japan) with nickel-filtered Cu-*K*α radiation (wave length λ = 1.5405 Å) and a high-speed one-dimensional detector (D/tex Ultra, Rigaku Corporation, Akishima, Japan) at a scanning rate of 5°·min^−1^. The crystallinity (*W*_c_, %) was evaluated according to the following formula [26,27]:

(2) $$W_{c}=\frac{I_{c}}{I_{c}+I_{a}}\times 100\%$$

where *I*_c_ and *I*_a_ are the scattering intensities of the crystalline region and amorphous region, respectively.

#### 2.4.4. Fourier Transform Infrared Spectrometer (FTIR)

The chemical composition and structure of copolymer materials were evaluated using a Fourier transform infrared spectrometer (FT/IR-460 Plus, JASCO International Co., Ltd., Tokyo, Japan). The spectra were recorded with 32 scans in transmittance mode with a resolution of 0.5 cm^–1^ within the range of 1000 to 3200 cm^–1^. 

## 3. Results and Discussion

### 3.1. Effect of Monomer Ratio

**Figure 2:**
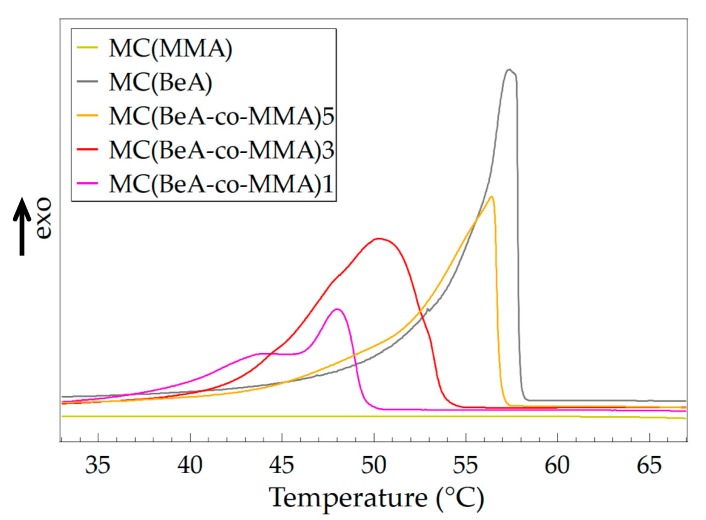
DSC curves of MC(BeA-*co*-MMA) copolymer microcapsules and MC(BeA) in the cooling process measured at a cooling rate of 5 °C·min^–1^.

The crystal transition behavior of microcapsules determines the thermal properties and energy storage capacities. Figure 2 shows the DSC curves of MC(BeA-*co*-MMA) microcapsules with an *n*-behenyl side-chain in the cooling process, measured at a cooling rate of 5 °C·min^–1^. The phase transition temperatures and phase change enthalpy are listed in Table 2. MC(BeA) had the strongest sharp exothermic peak with the highest phase change enthalpy. It indicated a quick transition from the isotropic phase to the crystal phase, and the great potential to be used as a thermal-energy-storage material. MC(MMA) was amorphous so it did not show any exothermic peaks in the cooling process. Compared to MC(MMA), MC(BeA-*co*-MMA) copolymer microcapsules showed an exothermic peak thanks to the introduction of the phase change component BeA. The peak was corresponding to crystallization of the *n*-behenyl side-chains. In the comb-like copolymer, eight or nine methylene units of the side-chain in the vicinity of the main-chain are amorphous. It is confirmed that the polymers of octadecyl and hexadecyl methacrylates crystallize with the terminal parts of 9–10 units and 7–8 units. [28,29]. In the cooling process, the segment motion of the crystalline *n*-behenyl side-chain was restricted by the rigid copolymer skeleton. Crystalline side-chains did not have enough mobility to adjust the steric conformation to allow all methylene units to arrange in crystals. Thus, only the terminal parts of the *n*-behenyl side-chain could form crystal regions, which led to a weaker and broader exothermic peak for MC(BeA-*co*-MMA) copolymer microcapsules.

When the monomer ratio of BeA and MMA was 5:1, the crystallization temperature was only about 1 °C lower than that of MC(BeA). Additionally, the average phase change enthalpy Δ*H* of Δ*H*_c_ and Δ*H*_m_ was 97.6 J·g^–1^ for MC(BeA-*co*-MMA)5 and 105.1 J·g^–1^ for MC(BeA-*co*-MMA)3, respectively. This indicated that the *n*-behenyl side-chain of MC(BeA-*co*-MMA) microcapsules could form a relatively perfect crystal region ensuring a high energy storage capacity. When the monomer ratio of BeA to MMA was 1:1, there existed two exothermic peaks. Many investigations on the crystallization behavior of *n*-alkane indicate that there are metastable rotator phases before the isotropic liquid completely converted into a crystalline solid [30,31]. Rotator phases pass through one or more than one rotator phases between the isotropic and crystal phase, due to the gradual breakdown of orientation order. As the amount of MMA was increasing, a stronger restriction was caused by the copolymer skeleton, which contributed to the peak appearing at 43 °C.

**Figure 3:**
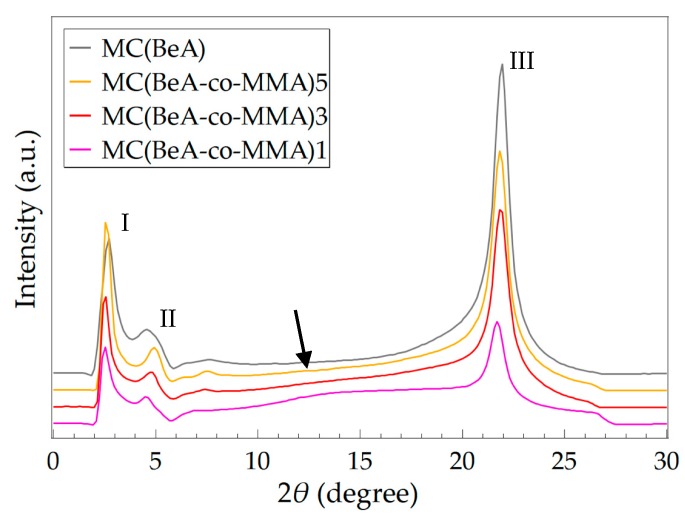
SAXS patterns of MC(BeA-*co*-MMA) microcapsules and MC(BeA) measured at room temperature. Three diffraction peaks were shown for both MC(BeA) and MC(BeA-*co*-MMA).

SAXS were carried out at room temperature for MC(BeA-*co*-MMA) microcapsules and MC(BeA), and the SAXS patterns are shown in Figure 3. The locations of diffraction peaks along with their corresponding interplanar crystal spacing *d* are listed in Table 3. MC(BeA-*co*-MMA) microcapsules could provide an energy storage capacity because of the crystal transition of the *n*-behenyl side-chain. Three peaks were observed at diffraction angles 2*θ =* 2.72°, 4.58° and 21.95° on the SAXS pattern for MC(BeA). The peak I at the small angle region was assigned to the layered structure of the alternating crystalline side-chain regions and amorphous regions. The interplanar crystal spacing *d* of Peak I and Peak II corresponded to the distance between copolymer chains separated by the side-chains. Researchers indicate that the long *n*-alkane chain compounds tend to crystallize into a lamellar structure, as the monolayer assemblies piled on one another, to arrange the functional groups regularly in each lamellar plane [28,32]. The peak I at 2.72° indicated the long spacing of 32.45 Å, which was nearly equal to the length of the fully extended BeA with a *n*-behenyl chain. It suggested that the side-chains of the comb-like copolymer seemed to be aligned perpendicularly to the basal plane. The intense diffraction Peak III at 2*θ =* 21.95° corresponded to the spacing of 4.05Å, which was considered the characteristic peak of the hexagonal packing of *n*-behenyl side-chains [14,32,33,34].

For MC(BeA-*co*-MMA) microcapsules, it was noted that with the increasing amount of BeA, diffraction Peak I and Peak II at small angles shift to higher angles, indicating a smaller interplanar crystal spacing *d*. This suggested that the peaking of the repeating units of the *n*-behenyl side-chain structure became denser with the decreasing amount of the amorphous component MMA. The PMMA segments with a pendent methyl group (–CH_3_) were hard, which prevented the copolymer chains from rotating freely around the carbon–carbon bond and packing closely in a crystal pattern. Whereas the interplanar crystal spacing (Bragg spacing) of Peak III at wide angles was almost the same and the value was close to that of MC(BeA), which revealed that the packing type of the *n*-behenyl side-chains was not affected by the monomer ratios. Besides, the addition of BeA caused the increasing intensity of Peak III. The increasing amount of BeA led to an increase in the number of methylene units in the crystals, which promised a better thermal energy storage capacity. For sample MC(BeA-*co*-MMA)1, there could be seen an amorphous halo around 2*θ* = 14° (indicated by the arrow in Figure 3), which was considered to originate from the correlation between the segregate side-chains and main-chains [14]. All the above results discovered that the increasing amount of BeA had a strong effect on the long spacing structure and the thermal-energy-storage capacity. 

### 3.2. Effect of Temperature Changing 

**Figure 4:**
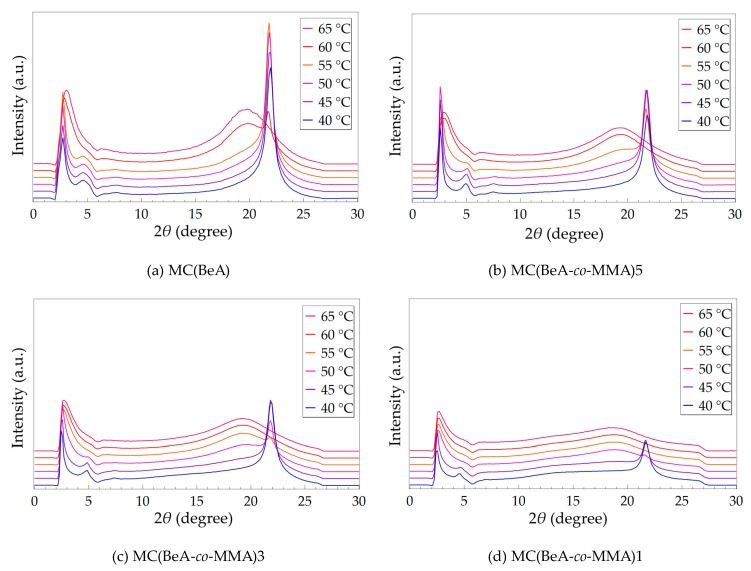
SAXS patterns of MC(BeA-*co*-MMA) microcapsules and MC(BeA) in the cooling process from the isotropic phase to the crystal phase at a cooling rate of 0.5 °C·min^−1^. The scale of intensity for each diagram is the same, aiming to give a clear understanding and direct comparison.

MC(BeA-*co*-MMA) microcapsules exhibit a complicated transition behavior between the isotropic phase and crystal phase. A temperature variable SAXS of MC(BeA-*co*-MMA) microcapsules and MC(BeA) were carried out in the cooling process at a slow rate of 0.5 °C·min^–1^ from 70 to 35 °C shown in Figure 4. In order to give a clear understanding and a direct comparison, the scale of intensity of each diagram is set to the same. The higher intensity and smaller half-peak width indicates a better crystal pattern with fewer disturbances from the main-chain skeleton [17,35]. For MC(BeA-*co*-MMA)5 and MC(BeA-*co*-MMA)3, the strong diffraction peaks suggested good crystallization properties, which ensured the high phase change enthalpy and good energy storage capacity. In our previous work, an infrared thermal testing was used to evaluate the thermoregulation property [18,20]. Owing to the good crystallization property, MC(BeA-*co*-MMA)5 and MC(BeA-*co*-MMA)3 effectively prolonged the time to reach the setting temperature, showing the huge capacity to store thermal energy.

MC(BeA-*co*-MMA) microcapsules showed a broad diffraction peak of the isotropic phase at 2*θ* = 19.82° for MC(BeA), 19.32° for MC(BeA-*co*-MMA)5, 19.31° for MC(BeA-*co*-MMA)3 and 18.38° for MC(BeA-co-MMA)1) caused by the disorder accumulation of the *n*-behenyl side-chains. Those strong peaks at the smaller angle of 2*θ =* 3.30° for MC(BeA), 2.84° for MC(BeA-*co*-MMA)5, 2.72° for MC(BeA-*co*-MMA)3 and 2.70° for MC(BeA-*co*-MMA)1) were also observed. Due to the tough and rigid property of PMMA segments, the interplanar spacing reduced with the decreasing amount of MMA. Therefore, the *n*-behenyl side-chains were found to transit into a crystal state at around 60 °C for MC(BeA-*co*-MMA)5, 55 °C for MC(BeA-*co*-MMA)3 and 50 °C for MC(BeA-*co*-MMA)1. These temperatures were close to the starting point of the corresponding exothermic peak of microcapsules according to DSC results. They showed that MC(BeA-*co*-MMA) microcapsules presented the exothermic peaks in the temperature range of 58 to 41 °C in the cooling process.

It is noted that within about 5 °C lower than the side-chain transition point, these peaks at the small angle area near 2*θ* = 4.5 to 5.0 appeared, which were close to the Peak II mentioned above in Figure 3. The peak corresponded to the long spacing order of copolymer chains separated by the side-chains. The copolymer main-chain structure could only transit into the solid phase at a lower temperature. In case of MC(BeA-*co*-MMA)1, the broad weak peak around 2*θ* = 14° appeared when the temperature cools down to 45 °C, which was similar to the temperature where the weak shoulder peak appeared on its DSC curve (Figure 2). It indicated that the correlation between the segregate side-chains and main-chains contributes to the phase transition. MMA units worked like defects in the side-chain crystal structure causing irregularity and reducing the flexibility of copolymer as a hard segment. As the amount of MMA decreases, less restriction was caused by the main-chain skeleton, and more methylene units of the *n*-behenyl side-chain could be arranged into crystals freely. This contributed to a higher phase change enthalpy and crystallinity of MC(BeA-*co*-MMA) microcapsules, so that provided a better energy storage capacity and thermoregulation property.

### 3.3. Effect of Temperature Changing Rate 

**Figure 5:**
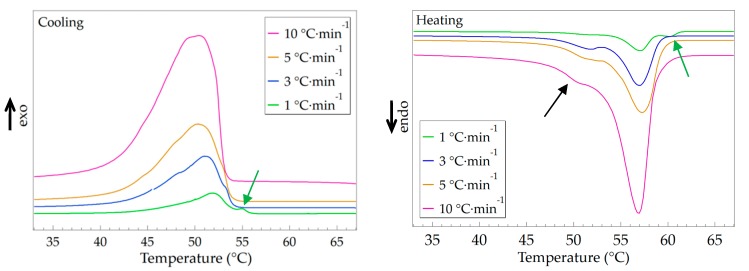
DSC curves of the MC(BeA-*co*-MMA)3 microcapsule measured at different temperature changing rates (including 1 °C·min^–1^, 3 °C·min^–1^, 5 °C·min^–1^ and 10 °C·min^–1^) in the cooling (left) and heating (right) processes.

The regularity of the repeating units strongly affects the crystal transition behavior of MC(BeA-*co*-MMA) microcapsules. DSC tests of MC(BeA-*co*-MMA)3 measured at different cooling/heating rates, including 1 °C·min^–1^, 3 °C·min^–1^, 5 °C·min^–1^ and 10 °C·min^–1^, were used to evaluate the crystal transition behavior. The relative DSC curves are shown in Figure 5, and the detailed data of the phase change temperature and phase transition enthalpy are listed in Table 4. In the temperature range of 40 to 60 °C, MC(BeA-*co*-MMA)3 displayed a different crystal transition behavior at various temperature changing rates. With the temperature changing rate increasing from 1 °C to 10 °C, the crystallization temperature tended to decrease; the same trend also for phase change enthalpy. On the cooling curve of MC(BeA-*co*-MMA)3 at the temperature changing rate of 1 °C·min^–1^, a small peak at 55 °C was detected, along with the corresponding endothermic peak at 60 °C in the heating process (indicated by the green arrows in Figure 5 (left) and Figure 5 (right), respectively). These peaks were not detected any more under the condition of a faster temperature changing rate. According to the XRD result of MC(BeA-*co*-MMA)3 shown in Figure 4c, the initial phase transition temperature of the crystalline *n*-behenyl side-chains was the same around 55 °C. The introduction of a tough and rigid PMMA segments reduced the flexibility of the copolymer chains and the mobility of the crystalline side-chains, and made the copolymer harder to form a crystal. On the heating curves of MC(BeA-co-MMA)3 at a higher temperature changing rate, a small shoulder endothermic peak was observed below the melting point (indicated by the black arrow in Figure 5 (right)). It revealed the existence of a metastable crystal forming below melting point with a faster cooling process. Melting of a remaining metastable crystal form occurred at a temperature near 48 °C, and then melting of the most stable form occurred.

**Figure 6:**
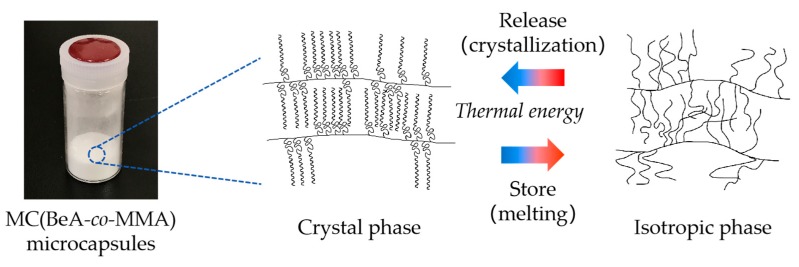
Schematic diagram of the phase transition behavior and crystal structure of MC(BeA-*co*-MMA) microcapsules with a crystalline *n*-behenyl side-chain.

As discussed in the previous section, the comb-like copolymer with a crystalline long *n*-alkane side-chain attached to the main-chain skeleton packed into a layered structure. The side-chains of the comb-like MC(BeA-*co*-MMA) microcapsules were aligned perpendicularly to the basal plane on the crystal pattern according to XRD results. The crystal structure of the MC(BeA-*co*-MMA) copolymer microcapsules with a crystalline *n*-behenyl side-chain are shown in Figure 6. Their crystal transition behavior can be interpreted as follows. At a temperature above the melting point, the copolymer was in the isotropic phase. As the temperature was going down, *n*-behenyl side-chains arranged into a crystal packing form, starting semi-crystalline and reaching a stable crystal pattern. Due to the restriction of the rigid copolymer skeleton, only the terminal parts of the *n*-behenyl side-chain could pack into crystals. The increasing amount of BeA allowed methylene units of the *n*-behenyl side-chain to arrange into crystals more freely, and contributed to a higher phase change enthalpy and crystallinity.

### 3.4. Effect of Synthesis Method

**Figure 7:**
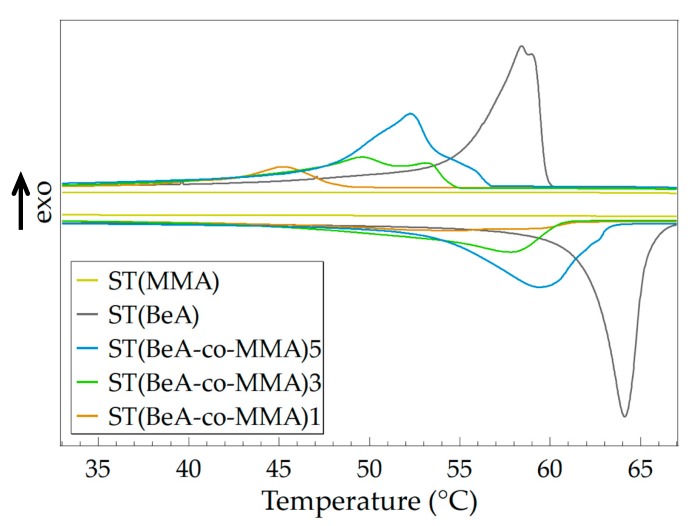
DSC curves of sheet samples for ST(BeA-*co*-MMA) copolymers with different monomer ratios and homopolymers ST(MMA) and ST(BeA) measured at a temperature changing rate of 5 °C·min^–1^.

By adjusting the monomer ratio of the copolymer, microcapsules with varying phase transition behavior and thermal properties can be prepared. Using copolymer microcapsules and sheets with the same ingredients as samples, we investigated the effects of synthesis method on the thermal and crystallization performance of the copolymer materials. Monomer BeA and MMA can form copolymer sheets under UV irradiation through free-radical light-induced polymerization. The DSC curves of ST(BeA-*co*-MMA) copolymer sheets are shown in Figure 7, and the detailed data are listed in Table 5. 

Like microcapsules, the phase change enthalpy and phase transition temperature of the copolymer sheets increased with the increasing amount of phase change component BeA. However, the phase transition behavior of copolymer sheets were different from that of microcapsules. Under the same monomer ratio, the copolymer sheet sample exhibited a lower phase change enthalpy, a lower phase transition temperature and a higher degree of supercooling. The microcapsule samples were synthesized through suspension polymerization, and the yield was around 60%. This indicated a loss of monomers during the polymerization process. Owing to the slight solubility of MMA in water, part of the MMA might dissolve in the water phase. In addition, the emulsion system in suspension polymerization was a little bit unstable, which might also cause a loss. On the other hand, the sheet samples were synthesized through light-induced polymerization. The mixture of monomer BeA, monomer MMA, crosslinker MBAA and initiator BP in a homogeneous phase was injected into a mold. The sheet samples were prepared after 48 h UV irradiation. There was no loss because the added resin was included in the sheet one hundred percent. Clearly, the content of MMA in sheet samples were higher. The larger amount of MMA caused a stronger restriction on the mobility of the crystalline *n*-behenyl side-chain, and formed more disturbances in the crystals. This reason explained the above DSC results in that the ST(BeA-*co*-MMA) copolymer sheets presented weak shoulder peaks and longer melting ranges (Figure 7).

**Figure 8:**
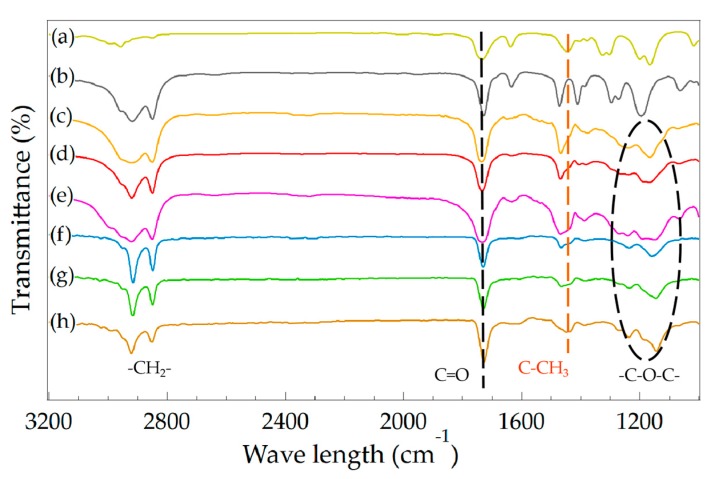
FTIR spectra of microcapsule and sheet samples for BeA-*co*-MMA copolymer and monomers MMA and BeA. (a) MMA, (b) BeA, (c) MC(BeA-*co*-MMA)5, (d) MC(BeA-*co*-MMA)3, (e) MC(BeA-*co*-MMA)1, (f) ST(BeA-*co*-MMA)5, (g) ST(BeA-*co*-MMA)3, and (h) ST(BeA-*co*-MMA)1.

The chemical composition and structure of MC(BeA-*co*-MMA) and ST(BeA-*co*-MMA) copolymer materials were evaluated by FTIR spectroscopy, presented in Figure 8. Thermal-energy-storage copolymers synthesized from different methods showed the similar spectrum profile containing a series of characteristic absorption. The copolymers showed strong peaks with double intensive absorption appearing at 2920 cm^–1^ and 2850 cm^–1^ owing to the alkyl C–H stretching vibrations of the methylene group. The C=O stretching vibration of the ester group triggered an absorption band near 1734 cm^–1^. The peaks near 1260, 1160 and 1130 cm^–1^, which could be assigned to the C–O stretching vibration of the ester group, were typical of acrylic ester. The absorption band at 1444 cm^–1^, which was attributed to the C–H bending vibration of the methyl group on the polymer backbone peak derived from MMA, were detected. Compared to microcapsules, these peaks of sheets at 1444 cm^–1^ were stronger, and the intensity became weaker with the increasing amount of BeA. It indicated the content of MMA in sheets was higher than that of microcapsules. It matched the DSC results.

**Figure 9:**
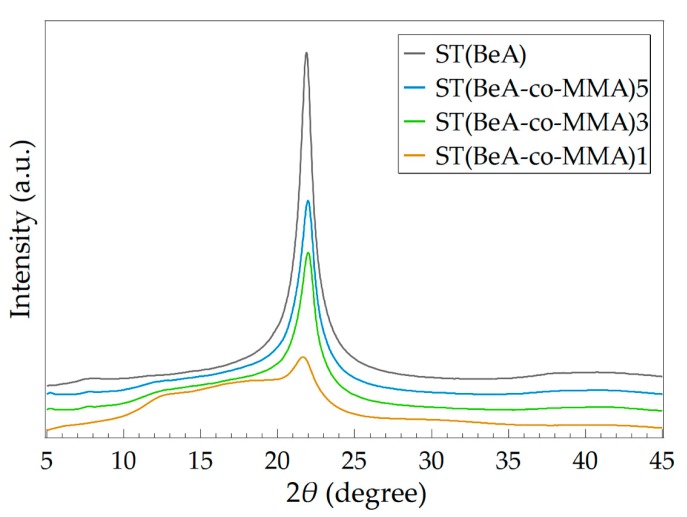
Wide-angle X-ray scatting (WAXS) patterns of ST(BeA-*co*-MMA) copolymer sheets and homopolymer ST(BeA) measured at room temperature.

The crystal structure of ST(BeA-*co*-MMA) copolymer sheets were further investigated by WAXS analysis. WAXS patterns of these copolymer sheets and homopolymer ST(BeA) are shown in Figure 9. The values of *W*_c_ and the location of the strongest peak with the corresponding interplanar crystal spacing *d* for them are listed in Table 6. Noted that despite synthesis methods were different, the diffraction peaks of the crystal packing of the *n*-behenyl side-chains were around 2*θ =* 21.86°, close to that of copolymer microcapsules. When the monomer ratio of BeA to MMA was 1:1, *W*_c_ was only 13.39% (Table 6), and the average Δ*H* of Δ*H*_c_ and Δ*H*_m_ was only 24.5 J·g^–1^ (Table 5). ST(BeA-*co*-MMA)1 could barely provide energy storage capacity because of relatively low Δ*H*. With the increasing amount of BeA, the crystallinity of copolymer sheets increased and the thermal energy storage capacity improved. The highest *W*_c_ and Δ*H* reached 38.23% and 91.0 J·g^–1^ under a monomer ratio of BeA to MMA at 5:1. The effect of synthesis method was mainly on the copolymer chemical component, but lightly on the crystal packing of the *n*-behenyl side-chains. ST(BeA-*co*-MMA) copolymer sheets exhibited complex crystal transition behavior, and the crystalline *n*-behenyl side-chains suffered a stronger restriction from the rigid copolymer skeleton during the crystallization process.

## 4. Conclusions

To figure out the correlation between the thermal properties and crystal structure of the thermal-energy-storage BeA-*co*-MMA copolymer, the effects of monomer ratio, temperature changing and the changing rate, as well as synthesis method, were investigated. Firstly, we synthesized MC(BeA-*co*-MMA) microcapsules through suspension polymerization. The monomer ratio influenced the crystal transition behavior and thermal properties greatly. The pendent *n*-behenyl group of BeA was highly crystalline, and it acted as a side-chain in the structure of the BeA-*co*-MMA copolymer. The highly crystalline *n*-behenyl side-chain provided copolymer the thermal-energy-storage capacity. The DSC results proved that when the monomer ratio of BeA and MMA was 3:1, MC(BeA-*co*-MMA)3 showed the highest average phase change enthalpy Δ*H* (105.1 J·g^–1^). This indicated that the *n*-behenyl side-chain could form a relatively perfect crystal region ensuring the high energy storage capacity. From the SAXS results, we knew that with the amount of BeA increasing, the interplanar crystal spacing *d* at small angles became smaller, while the interplanar crystal spacing (Bragg spacing) at wide angles remained almost unchanged. All the above DSC and SAXS results proved that the amount of BeA had a strong effect on the thermal-energy-storage capacity of the copolymer and the long spacing of crystals, but barely on the crystal lamella. We also discussed the effects of temperature changing rate. The results clarified that MMA units worked like defects in the *n*-behenyl side-chain crystal structure of the BeA-*co*-MMA copolymer. With the decreasing amount of MMA, less restriction was caused by the copolymer skeleton, and more methylene units of the *n*-behenyl side-chain could arrange into crystals freely. That is, a higher fraction of BeA contributed to the higher crystallinity of the MC(BeA-*co*-MMA) copolymer, which promised a better energy storage capacity and thermoregulation property. The influence of synthesis method on the crystal transition behavior and thermal properties was investigated by comparing ST(BeA-*co*-MMA) copolymer sheets prepared through light-induced polymerization with the same ingredients as in MC(BeA-*co*-MMA). We found that the effect of synthesis method was mainly on the copolymer chemical component, but lightly on the crystal packing of the *n*-behenyl side-chains.

## Figures and Tables

**Table 1:** Composition of all the thermal-energy-storage copolymer microcapsules and sheets with a crystalline *n*-behenyl side-chain synthesized in this work by varying monomer mass ratios.

Sample		Synthesis Method	Monomers	Monomer Ratio
Microcapsule	MC(MMA)	Heat-initiated suspension polymerization	MMA	–
	MC(BeA)		BeA	–
	MC(BeA-*co*-MMA)1		BeA:MMA	1:1
	MC(BeA-*co*-MMA)3		BeA:MMA	3:1
	MC(BeA-co-MMA)5		BeA:MMA	5:1
Sheet	ST(MMA)	Light-induced polymerization	MMA	–
	ST(BeA)		BeA	–
	ST(BeA-*co*-MMA)1		BeA:MMA	1:1
	ST(BeA-*co*-MMA)3		BeA:MMA	3:1
	ST(BeA-*co*-MMA)5		BeA:MMA	5:1

**Table 2:** Thermal properties of MC(BeA) and MC(BeA-*co*-MMA) copolymer microcapsules with different monomer ratios, measured at a temperature changing rate of 5 °C·min^−1^.

Sample	Crystallization		Melting		Degree of Supercooling (°C)
	*T*_c_ (°C)	Δ*H*_c_ (J·g^–1^)	*T*_m_ (°C)	Δ*H*_m_ (J·g^–1^)	
MC(MMA)	-	-	-	-	-
MC(BeA)	57.4	115.6	61.8	114.3	4.4
MC(BeA-*co*-MMA)5	56.4	98.2	61.8	97.0	5.4
MC(BeA-*co*-MMA)3	50.4	105.2	57.3	104.9	6.9
MC(BeA-*co*-MMA)1	48.0	56.1	54.1	56.5	6.1

*T*_c_: crystallization temperature; Δ*H*_c_: enthalpy of crystallization process; *T*_m_: melting temperature; Δ*H*_m_: enthalpy of melting process; Degree of supercooling: temperature difference between *T*_m_ and *T*_c_ (Δ*T = T*_m_ − *T*_c_). Enthalpy values during crystallization and melting can be considered identical within instrumental errors.

**Table 3:** The locations of diffraction peaks and corresponding interplanar crystal spacing *d* of MC(BeA-*co*-MMA) microcapsules and MC(BeA) calculated from the SAXS results.

Sample	Peak I (2θ)	*d*_1_ (Å)	Peak II (2θ)	*d*_2_ (Å)	Peak III (2θ)	*d*_3_ (Å)
MC(BeA)	2.72	32.45	4.58	19.28	21.95	4.05
MC(BeA-*co*-MMA)5	2.55	34.62	4.93	17.91	21.82	4.07
MC(BeA-*co*-MMA)3	2.44	36.18	4.86	18.17	21.82	4.07
MC(BeA-*co*-MMA)1	2.40	36.78	4.49	19.67	21.69	4.09

**Table 4:** Thermal properties of the MC(BeA-*co*-MMA)3 microcapsule measured at different temperature changing rates (including 1 °C·min^–1^, 3 °C·min^–1^, 5 °C·min^–1^ and 10 °C·min^–1^).

Temperature Changing Rate	Crystallization		Melting	
	*T*_c_ (°C)	Δ*H*_c_ (J·g^–1^)	*T*_m_ (°C)	Δ*H*_m_ (J·g^–1^)
1 °C·min^–1^	51.8	112.5	57.1	114.7
3 °C·min^–1^	51.1	105.8	56.9	106.3
5 °C·min^–1^	50.4	105.2	57.3	104.9
10 °C·min^–1^	50.3	102.1	56.9	103.1

**Table 5:** Thermal properties of sheet samples measured at a temperature changing rate of 5 °C·min^–1^ for the ST(BeA-*co*-MMA) copolymer with different monomer ratios and homopolymers ST(MMA) and ST(BeA).

Sample	Crystallization		Melting		Degree of Supercooling (°C)
	*T*_c_ (°C)	Δ*H*_c_ (J·g^–1^)	*T*_m_ (°C)	Δ*H*_m_ (J·g^–1^)	
ST(MMA)	-	-	-	-	-
ST(BeA)	58.4	115.2	64.1	115.9	5.7
ST(BeA-*co*-MMA)5	52.3	91.0	59.4	91.0	7.1
ST(BeA-*co*-MMA)3	49.7	61.6	57.8	61.4	8.1
ST(BeA-*co*-MMA)1	45.2	23.7	55.0	25.2	9.8

**Table 6:** The location of diffraction peak with its corresponding interplanar crystal spacing *d*, and crystallinity (*W*_c_) of ST(BeA-*co*-MMA) copolymer sheets and homopolymer ST(BeA) measured at room temperature.

Sample	Peak (2*θ*)	*d* (Å)	*W*_c_ (%)
ST(BeA)	21.86	4.06	56.67
ST(BeA-*co*-MMA)5	21.98	4.04	38.23
ST(BeA-*co*-MMA)3	21.98	4.04	29.78
ST(BeA-*co*-MMA)1	21.69	4.10	13.39
